# Screening protocol for dysphagia in adults: comparison with videofluoroscopic findings

**DOI:** 10.6061/clinics/2017(12)01

**Published:** 2017-12

**Authors:** Fernanda C. Sassi, Gisele C. Medeiros, Bruno Zilberstein, Shri Krishna Jayanthi, Claudia R.F. de Andrade

**Affiliations:** I Departamento de Fisioterapia, Fonoaudiologia e Terapia Ocupacional, Faculdade de Medicina (FMUSP), Universidade de Sao Paulo, Sao Paulo, SP, BR; II Divisão de Fonoaudiologia, Hospital das Clínicas HCFMUSP, Faculdade de Medicina, Universidade de Sao Paulo, Sao Paulo, SP, BR; III Divisao de Cirurgia Digestiva, Hospital das Clinicas HCFMUSP, Faculdade de Medicina, Universidade de Sao Paulo, Sao Paulo, SP, BR; IV Instituto de Radiologia, Hospital das Clinicas HCFMUSP, Faculdade de Medicina, Universidade de Sao Paulo, Sao Paulo, SP, BR

**Keywords:** Dysphagia, Screening Test, Modified Barium Swallow, Aspiration, Pneumonia

## Abstract

**OBJECTIVES:**

To compare the videofluoroscopic findings of patients with suspected oropharyngeal dysphagia with the results of a clinical screening protocol.

**METHODS:**

A retrospective observational cohort study was conducted on all consecutive patients with suspected oropharyngeal dysphagia between March 2015 and February 2016 who were assigned to receive a videofluoroscopic assessment of swallowing. All patients were first submitted to videofluoroscopy and then to the clinical assessment of swallowing. The clinical assessment was performed within the first 24 hours after videofluoroscopy. The videofluoroscopy results were analyzed regarding penetration/aspiration using an 8-point multidimensional perceptual scale. The accuracy of the clinical protocol was analyzed using the sensitivity, specificity, likelihood ratios and predictive values.

**RESULTS:**

The selected sample consisted of 50 patients. The clinical protocol presented a sensitivity of 50% and specificity of 95%, with an accuracy of 88%. “Cough” and “wet-hoarse” vocal quality after/during swallowing were clinical indicators that appeared to correctly identify the presence of penetration/aspiration risk.

**CONCLUSION:**

The clinical protocol used in the present study is a simple, rapid and reliable clinical assessment. Despite the absence of a completely satisfactory result, especially in terms of the sensitivity and positive predictive values, we suggest that lower rates of pneumonia can be achieved using a formal dysphagia screening method.

## INTRODUCTION

Dysphagia is defined as difficulty in swallowing foods, liquids or both 1. Neurological, muscular, anatomical, and/or psychological factors may predispose a person to present difficulty in swallowing 2. The prevalence of functional oropharyngeal dysphagia is very high; it affects more than 37%-78% of acute ischemic and hemorrhagic stroke patients 3,4, 52-82% of patients with neurodegenerative diseases, 42-87% of patients after prolonged orotracheal intubation 5, more than 35% of patients with head and neck diseases 6, and more than 60% of elderly institutionalized patients 7.

The movements involved in the act of swallowing are intended not only to obtain nourishment but also to protect the respiratory tract 8. Thus, underlying clinical conditions may interact with dysphagia to produce aspiration, pneumonia, and/or respiratory compromise 9. Moreover, dysphagia may interfere with nutrition, delay clinical recovery and even lead to death if not diagnosed early and properly 1,10. Therefore, earlier detection of dysphagia leads to earlier selection of an adequate treatment. This not only shortens the reestablishment of the overall health status (i.e., a return to effective swallowing function and nutritional homeostasis) but also reduces the overall rehabilitation costs 11,12.

Currently, no consensus exists on a standard method of assessment 13. Screening tools and bedside swallowing assessments are routinely conducted as initial assessments of swallowing, after which an instrumental evaluation may be performed 14. Bedside screening of dysphagia has been shown to effectively reduce the incidence rate of pneumonia, and dysphagia screening has been gradually incorporated into guidelines for the care of specific groups of patients 15,16. These instruments, however, have variable sensitivities and specificities for detecting oropharyngeal dysphagia and aspiration 17-20. To date, the Toronto Bedside Swallowing Screening Test (TOR-BSST) reported in a study by Martino et al. 21 is the only instrument that has shown high sensitivity and high negative predictive values for the early detection of dysphagia. Unfortunately, this protocol has only been validated for stroke patients.

Videofluoroscopy (VFS), also called a modified barium swallow study, is the gold standard method for studying the oral and pharyngeal mechanisms of dysphagia and for evaluating the efficacy and safety of swallowing 7. VFS provides direct visualization of the anatomy and physiology of swallowing, including the motions of the jaws, tongue, palate, pharynx, larynx, and esophagus 22. Although VFS is considered the criterion standard for identifying aspiration/silent aspiration during swallowing, it generally incurs a substantial monetary cost, entails radiation exposure, has limited standardization, and is not feasible for all patients (e.g., moderately ill patients cannot be transported to the radiology department) 23. In addition, VFS requires specialized equipment and personnel who are not readily available in many hospitals 24.

This study was specifically designed to compare the videofluoroscopic findings of patients with suspected oropharyngeal dysphagia with the results of a clinical screening protocol.

## MATERIALS AND METHODS

The study was approved by an institutional review board to ensure the ethical conduct of research studies with human subjects (CAPPesq HCFMUSP 1.781.177). Written informed consent was obtained from all participants.

### Study Participants

We conducted a retrospective observational cohort study. The study population included all consecutive patients with suspected oropharyngeal dysphagia who were referred by the medical team to the Radiology Institute of *Hospital das Clínicas* to complete a videofluoroscopic assessment of swallowing between March 2015 and February 2016. Patients were eligible if they met the following criteria: a) older than 18 years; b) ≥14 points on the Glasgow Coma Scale; c) absence of a tracheostomy tube; d) no medical contraindications to performing a barium swallow due to radiation exposure, allergies or postural limitations; e) no restrictions on liquid intake; and f) completion of the clinical assessment of swallowing within the first 24 hours after VFS.

### Measures

The swallowing assessment was performed in two parts:

Part 1 - Videofluoroscopic assessment of swallowing

All patients first completed VFS. The fluoroscopy unit used in this study was the GE Medical Systems ADVANTX (GE Healthcare, Wakeska, Wisconsin, USA). All VFS studies were performed in the lateral plane by a VFS-trained radiographer and two trained speech and language therapists. The participants remained seated, at an angle of 90°, with their heads positioned horizontally during the entire examination. Liquid barium (Opti-bar) at a concentration of 100% w/v was used. The protocol adopted for the swallowing assessment involved the ingestion of food with different consistencies and is routinely used at our hospital to investigate swallowing characteristics, especially the presence of aspiration. In the present study, we only considered the swallowing of 10 ml of the liquid consistency (liquid barium at a concentration of 100% w/v) for analysis. The mixture was measured using a disposable syringe and was offered to the participants in a cup.

Swallowing was analyzed by reviewing the digitalized images of each swallow. Penetration/aspiration 25 was determined using an 8-point multidimensional perceptual scale. Scores were assigned as follows: 1 - material does not enter the airway; 2 - material enters the airway, remains above the vocal folds, and is ejected from the airway; 3 - material enters the airway, remains above the vocal folds and is not ejected from the airway; 4 - material enters the airway, touches the vocal folds and is ejected from the airway; 5 - material enters the airway, touches the vocal folds and is not ejected from the airway; 6 - material enters the airway, passes below the vocal folds and is ejected into the larynx or out of the airway; 7 - material enters the airway, passes below the vocal folds and is not ejected from the trachea despite effort; 8 - material enters the airway, passes below the vocal folds, and no effort is made to eject it. Patients were diagnosed with dysphagia if they received a score of 4 points or above on the perceptual scale.

Despite the acceptance of this technique as the gold standard for evaluating swallowing abilities, its reliability among experts remains low 26. Thus, two speech-language pathologists who were not involved in performing the swallowing study, each with more than four years of experience with dysphagia, reviewed each VFS result. The interrater reliability was high, with an intraclass correlation coefficient (ICC) of 0.90.

Part 2 – Clinical assessment of swallowing

The clinical assessment of swallowing was performed within the first 24 hours after VFS and involved the application of the Dysphagia Risk Evaluation Protocol (DREP) 9,27. This protocol is routinely used by the Division of Orofacial Myology at our hospital to assess swallowing dysfunction in patients. The protocol includes items previously described as being effective in identifying high-risk patients with suspected dysphagia 28,29. Items were chosen based on several guiding principles, such as ease of administration, ease of interpretation and pre-existing research supporting each item’s relationship with dysphagia. The protocol is divided into two sections, a water swallow test and a puree/solid swallow test, and the results are marked as either pass or fail for each of the observed items. Based on a previous study 27, we only used the results obtained from the water swallow test and analyzed the variables considered as possibly significant high-risk indicators of dysphagia (i.e., multiple swallows, cervical auscultation, vocal quality, cough and choking).

As determined by the authors of the protocol, patient swallowing was assessed during the administration of 5 ml of water (via a syringe). The test was repeated, if necessary, up to 3 times to confirm the results. Patients were placed in an upright position so that their sitting position did not interfere with the research results. The assessed items and the criteria used to interpret the results were as follows:

- Multiple swallows per bolus: Pass - The patient only requires only 1 swallow per bolus. Fail - The patient presents more than one swallow per bolus, presents drooling/spillage from mouth, or requires cues to complete the task.
- Cervical auscultation (a stethoscope is placed at the lateral aspects above the cricoid cartilage and in front of the sternocleidomastoid muscle and large vessels): Pass - The patient presents the 3 characteristic sounds (two clicks followed by an expiratory sound), indicating that the bolus has passed through the pharynx. Fail - The patient does not present any sound or presents sounds other than those described above.
- Vocal quality: Pass - The patient does not present any alterations within the first minute after swallowing. Fail - The patient’s voice becomes gurgly (“wet”) within the first minute after swallowing.
- Cough: Pass - The patient does not cough within the first minute after swallowing. Fail - The patient coughs (voluntary or not) with or without throat clearing within the first minute after swallowing.
- Choking: Pass - The patient does not choke after swallowing. Fail - The patient chokes during and/or after swallowing.

Patients were considered as being at risk for penetration/aspiration if they failed at least one of the above items. The only exception was for the item “multiple swallows”, as reports in the literature have shown that this sign can be related to a physiological adaptation and therefore cannot be interpreted as an alteration in the swallowing mechanism 30.

### Data Analysis

Univariate statistics were used to describe the data, including percentages, means and standard deviations. The accuracy of the clinical protocol was tested using the sensitivity, specificity, likelihood ratios, and predictive values. The agreement between the VFS and clinical protocol results was verified using Cohen’s kappa coefficient.

## RESULTS

The selected sample consisted of 50 patients (13 males and 37 females), with ages ranging from 24 to 87 (62.2±16.0) years. Patients were diagnosed with the following medical conditions: 20 (40%) had gastroenterological diseases, 15 (30%) had neurological diseases, 8 (16%) had pulmonary diseases, 4 (8%) had cardiologic diseases, 2 (4%) had vascular diseases, and 1 (2%) had rheumatologic disease.

**Figure 1:**
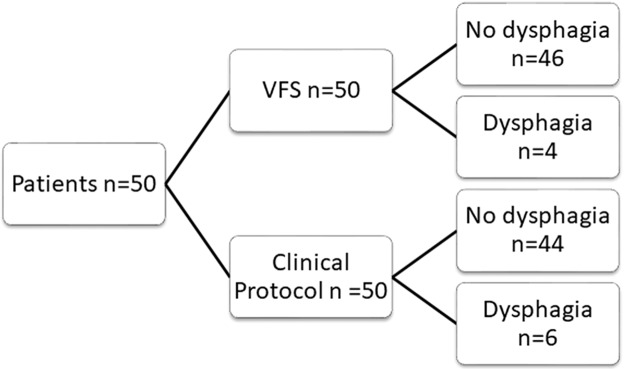
Flow diagram for the swallowing study. n – number of patients, VFS – videofluoroscopy of swallowing.

Figure 1 shows the results obtained from VFS and the clinical protocol.

Tables 1 and 2 display the distribution of patients according to their results on VFS and the clinical protocol, respectively. According to the results of both instruments, most patients presented normal swallowing function. Patients who had altered VFS results exhibited an even distribution among the scores, which was considered as altered on the multidimensional scale. Regarding the clinical signs, most patients who failed presented cough followed by alterations in vocal quality.

A comparison of the results obtained for the instruments used to identify dysphagia is presented in Table 3. The clinical protocol had a sensitivity of 50% and a specificity of 91.3% compared to VFS. The negative predictive value was 95%, with an accuracy of 88%. Cohen’s kappa coefficient indicated an agreement of 0.336 between the tests, i.e., a fair agreement. For the two participants who had indications of dysphagia on both instruments (true positive results), the most effective predictor was the presence of cough (100%).

## DISCUSSION

Dysphagia screening tools are the most widely used methods of assessing oropharyngeal dysphagia worldwide 8,14. The tests are typically easy to perform and extremely useful for obtaining a rough estimation of the swallowing condition 8. Evidence shows that the implementation of screening tools to identify risks of dysphagia has resulted in substantial reductions in pneumonia rates 15,16. Moreover, the literature suggests that protocols including a water swallow test yield the best patient outcomes 15,31. In this sense, the DREP fits with what has previously been described in the literature as a good bedside swallowing assessment for dysphagia.

Although aspiration is not present in all dysphagia patients, it is considered the most important symptom associated with swallowing dysfunction 8. During the assessment of dysphagia, choking is a conspicuous symptom that can be objectively observed. However, a study by Wu et al. 32 reported that choking is a poor indicator of aspiration because it cannot predict silent aspiration. In our study, of 6 patients who failed the DREP, 5 patients presented coughing after the water swallow test, and 1 presented an altered vocal quality (i.e., a wet-hoarse voice). Our findings are consistent with previous studies that have reported both items as being sensitive for detecting penetration/aspiration 17,33.

Regarding accuracy, the DREP demonstrated a high specificity and negative predictive value but only a moderate sensitivity: 50% for aspiration/penetration. The specificity of the DREP is at the higher end of the range of that of other dysphagia screening tests, which varies from 49% to 67% 19,31. In the present study, VFS showed penetration/aspiration to be present in 4 patients. A comparison of the ability of the DREP and VFS to detect penetration/aspiration revealed that the DREP correctly identified penetration/aspiration in 2 patients who also presented altered results on VFS. For the remaining 2 patients who presented altered results on VFS, the DREP indicated false negative results. Moreover, the DREP yielded 4 false positive results, indicating that the screening protocol may have a tendency to over-identify penetration/aspiration. Poor sensitivity values (i.e., 47%) 34 and overestimation of the risk for penetration and aspiration have previously been reported in the literature 14,35. The number of false positive results of the DREP does not seem to have a major negative impact on patients, as it may only lead to a VFS referral. However, the necessity for a more specific assessment involves additional costs and is usually time consuming 21.

In our study, selection bias primarily accounted for the relatively moderate sensitivity and low positive predictive values of the DREP in identifying swallowing dysfunction. According to the criteria adopted by our hospital (i.e., public, high complexity, limited staff with expertise to perform VFS), patients who are suspected of having dysphagia are first referred for complete VFS by the medical team, who in many cases do not have the necessary knowledge to identify swallowing disorders, and then to a speech-language pathologist for a clinical assessment. Consequently, 46 patients were diagnosed as having no dysphagia according to the gold standard parameter. The diagnosis and management of dysphagia requires a multidisciplinary approach 6. As is the case with other pathological conditions, the literature suggests that a diagnostic procedure should start with a screening test 8. Based on the screening results, only high-risk patients who are reasonably suspected of having dysphagia should proceed to a more specific examination (i.e., VFS or video endoscopic examination). This procedure avoids not only radiation exposure, but also unnecessary costs.

Notably, one central aim of our study was to determine the specificity and sensitivity of using a clinical protocol to detect penetration/aspiration in a heterogeneous group of patients. Most of the existing screening methods have only been validated in patients with dysphagia caused by stroke 3,16,17,20,21,34, which limits the application of these tests to patients with dysphagia caused by other diseases. Despite the absence of a completely satisfactory result, especially in terms of sensitivity and positive predictive values, we suggest that lower rates of pneumonia can be achieved using a formal dysphagia screening method 15.

Our study should be viewed as an effort to standardize care for patients with swallowing disorders across all settings. Given the current trend of having an evidence-based method 9, screening tools validated in different populations with dysphagia will allow for earlier referral to properly diagnose a patient and direct treatment, which will reduce complications, malnutrition and even death. Future studies will involve the application of the DREP prior to VFS in specific populations using different food consistencies and volumes for possible adjustments.

## AUTHOR CONTRIBUTIONS

Sassi FC organized and conducted the statistical analyses, interpreted the results and wrote a major portion of the manuscript. Medeiros GC collected and analyzed data, interpreted the results and wrote the manuscript. Zilberstein B participated in the data analyses, interpretation of the results and writing of the manuscript. Jayanthi SK participated in the data analyses, interpretation of the results and writing of the manuscript. de Andrade CR was responsible for the research and experimental design, contributed to the data analysis and manuscript preparation.

## Figures and Tables

**Table 1:** Distribution of patients according to the multidimensional perceptual scale (videofluoroscopy of swallowing).

Score	n	%
1	31	62
2	4	8
3	11	22
4*	1	2
5	1	2
6	0	0
7	1	2
8	1	2

n – number of participants; % – percentage of participants;

* cut-off value for dysphagia.

**Table 2:** Distribution of patients according to the clinical protocol.

Clinical signs		n (%)
Multiple swallows	Pass	29 (58)
	Fail	21 (42)
Cervical auscultation	Pass	50 (100)
	Fail	0
Voice quality	Pass	49 (98)
	Fail	1 (2)
Cough	Pass	45 (90)
	Fail	5 (10)
Choking	Pass	50 (100)
	Fail	0

n – number of participants; % – percentage.

**Table 3:** Comparison of the accuracy measures of the clinical and VFS results.

		VFS		
		Dysphagia (n)	No dysphagia (n)	
Clinical protocol	Dysphagia (n)	2	4	PPV = 0.33
	No dysphagia (n)	2	42	NPV = 0.95
				PLR = 0.57
		Sensitivity = 50%	Specificity = 91.3%	NLR = 0.55
		False negative = 4%	False positive = 8%	

VFS – videofluoroscopy of swallowing; n – number of patients; PPV – positive predictive value; NPV – negative predictive value; PLR – positive likelihood ratio; NLR – negative likelihood ratio.
